# The application of radionuclide therapy for breast cancer

**DOI:** 10.3389/fnume.2023.1323514

**Published:** 2024-01-10

**Authors:** Anna Musket, Sandra Davern, Brianna M. Elam, Philip R. Musich, Jonathan P. Moorman, Yong Jiang

**Affiliations:** ^1^ Department of Internal Medicine, Quillen College of Medicine, East Tennessee State University, Johnson City, TN, United States; ^2^ Center of Excellence in Inflammation, Infectious Disease and Immunity, Quillen College of Medicine, East Tennessee State University, Johnson City, TN, United States; ^3^ Oak Ridge National Laboratory, Oak Ridge, TN, United States; ^4^ Department of Biomedical Science, Quillen College of Medicine, East Tennessee State University, Johnson City, TN, United States

**Keywords:** radionuclide, early diagnosis, breast cancer, tumor, therapy

## Abstract

Radionuclide-mediated diagnosis and therapy have emerged as effective and low-risk approaches to treating breast cancer. Compared to traditional anatomic imaging techniques, diagnostic radionuclide-based molecular imaging systems exhibit much greater sensitivity and ability to precisely illustrate the biodistribution and metabolic processes from a functional perspective in breast cancer; this transitions diagnosis from an invasive visualization to a noninvasive visualization, potentially ensuring earlier diagnosis and on-time treatment. Radionuclide therapy is a newly developed modality for the treatment of breast cancer in which radionuclides are delivered to tumors and/or tumor-associated targets either directly or using delivery vehicles. Radionuclide therapy has been proven to be eminently effective and to exhibit low toxicity when eliminating both primary tumors and metastases and even undetected tumors. In addition, the specific interaction between the surface modules of the delivery vehicles and the targets on the surface of tumor cells enables radionuclide targeting therapy, and this represents an exceptional potential for this treatment in breast cancer. This article reviews the development of radionuclide molecular imaging techniques that are currently employed for early breast cancer diagnosis and both the progress and challenges of radionuclide therapy employed in breast cancer treatment.

## 1 Introduction

A radionuclide is a nuclide carrying excess nuclear energy that can emit either *alpha* particles carrying high energy with a short range, *beta* particles carrying low energy with a longer range, *gamma* radiation with a long range, or *auger electrons* with a very short range and low energy (1). Amongst these, alpha particles and beta particles are the most popular choice for radionuclides in breast cancer (BC) diagnosis and treatment (2–4). These two forms of radiation energy can be safely harnessed to break down the physiological processes, most notably the genomic integrity of cancer cells, and eventually lead to cancer cell death and subsequent tumor shrinkage (2). Radionuclide therapy, as a major type of radiopharmaceutical therapy, started in November 1938 when John Lawrence used radioactive phosphorus (^32^P) to treat leukemia (5). However, radionuclide therapy for use on solid tumors had begun as early as the middle of the 19th century, not long after Dr. Marie Curie discovered radium which was found to damage live tissues and cells following exposure. Subsequently, radium has been employed to treat a variety of diseases besides cancer (6–11). Numerous clinical cases of radionuclide therapy have proved that it is a safe and effective approach for the treatment of many types of cancer (12–14).

Radionuclides are elements, for the medical field this typically means only those radionuclides from the actinide series on the table of elements, that emit energy in the form of radiation (15). The loss of this energy turns the radionuclide from one element to another (daughter isotopes) through a process called decay. In alpha decay, a composite particle composed of two protons and two neutrons is emitted that mimics helium in atomic mass, but it differs from helium in that it is double-ionized. There are two forms of beta particles emitted in beta decay and beta plus decay (also called positron emissions). In beta decay, electrons are expelled from the nucleus at high speeds; these electrons are termed beta particles and are typically used in radionuclide therapies. In beta plus decay, positrons are expelled from the nucleus, also at speeds close to the speed of light, and these positrons are commonly used in the imaging of disease states. Gamma rays are emitted from the nucleus in the form of electromagnetic energy as photons that can be used for either treatment or imaging modalities. Gamma-ray emissions can travel the greatest distance of the radiation types discussed herein. Another form of radioactive decay is electron capture, where orbital electrons fill in a vacancy in the nucleus, resulting in auger emissions. However, the emission of auger electrons requires a vacancy in the nucleus of the radionuclide, which can be induced by other forms of radioactive decay or excitation by an external force, for example by x-rays (16). Regardless of the form of decay, radionuclides express energy from the nucleus (called E_max_) that is responsible for cellular DNA damage. The level of energy, however, differs vastly in both the rate of decay and the damage potential.

Radionuclides can damage DNA through either direct or indirect damage. Direct damage can be complex DNA double-strand breaks, single-strand breaks, base damage, and cross-linkage formations (17). Indirect DNA damage typically arises through the generation of reactive species (oxygen or nitrogen) in the treated area that affects the cellular DNA to form mismatches, single-strand breaks, and double-strand breaks (17). Among the three types of radionuclides, alpha is the most ionizing with the greatest potential for direct DNA damage that does not rely on nearby reactive species generation (18, 19). Beta and gamma radionuclides, on the other hand, have less ionizing potential but can damage the DNA through the generation of reactive oxygen species and free radical species. The alpha particles also have a very high linear energy transfer, which contributes to the increased DNA damage potential by causing increasingly complex DNA lesions (20). Additionally, DNA double-strand breaks caused by high linear energy transfer radionuclides are repaired more slowly than are double-strand breaks caused by low linear energy transfer radionuclides (19). Beta particles emitting radionuclides, however, can frequently also generate gamma emissions as a side product of beta decay. Both the alpha and beta particles emitted from radionuclides have direct linear penetration, albeit the particles do not traverse great distances in tissues. However, beta particles can travel farther through the air, and gamma emissions are emitted as photons that travel in a wave that can sometimes co-occur with radionuclides undergoing beta decay. Comparatively, auger emissions have much lower energy and thus a shorter range of penetration in tissues of only a few hundred nanometers. For this reason, auger emitters must be incorporated into the nucleus of a cell to be effective in damaging DNA as a treatment modality.

Recently, targeted radionuclide therapy (TRT) has emerged as a promising strategy to significantly improve radiopharmaceutical efficiency while minimizing toxicity and other side effects (21–28). Unlike traditional radiation therapy in which administration occurs through an external beam, the radionuclides employed in therapy are administered intravenously, intraperitoneally, or orally. During the process of an ideal TRT, internally administered radionuclides will migrate specifically to the tumor region to precisely exert their cytotoxicity without any significant detrimental effects on the surrounding normal tissues (29, 30), as exemplified by a famous case of administration of ^131^I to patients with thyroid carcinoma (31–35). Specific targeting can be achieved through antigen-antibody recognition (36, 37), ligand-receptor interaction (38), or the interaction between certain biomolecules and unique biomarkers on the surface of tumor cells based on their high affinity (22). As such, the required dosage of radionuclide used in the treatment can be much less, which will significantly minimize the unnecessary exposure of patients to radiation both temporally and spatially. Also, TRT can significantly decrease the background radiation activity which may lead to high drug tolerance (39). In addition, radionuclides can emit either x-rays, gamma-rays, or beta-particles that can be visualized by nuclear medicine imaging systems, such as single-photon emission computed tomography (SPECT) scanning or PET, to directly monitor the efficacy and precision of TRT (40–42).

Accumulated clinical evidence has demonstrated the great treatment potential for TRT targeting both primary tumors and metastases, thus TRT presents a highly effective, safer, and more economical modality when compared to traditional chemotherapy (43). This review will cover the existing TRT in BC and will discuss the clinical development and challenges of TRT.

## 2 Radionuclide diagnosis in breast cancer

Breast cancer is the most frequent cancer occurring in women in the United States. According to statistical analyses from the American Cancer Society, about 290 thousand women were diagnosed with BC in 2022, rendering it the most common cancer in U.S. women. Unfortunately, more than 43,000 women died from BC during that year (44). Thus, robust and effective diagnostic and treatment regimens will be critical to improve outcomes for BC patients. As to BC diagnosis, radionuclide molecular imaging has demonstrated indisputable advantages over traditional anatomical imaging strategies that rely on finding the altered anatomical structure of breast tumors, such as mammography, ultrasound, magnetic resonance imaging (MRI), and computed tomography (CT) (45). BC is a highly heterogeneous disease with many subtypes according to its genetic and clinical background (46, 47). The most common classification of BC is based on the expression of estrogen receptor (ER), progesterone receptor (PR), and human epidermal growth factor receptor 2 (HER2). The expression of each of these receptors can be detected by immunohistochemical analyses. The four intrinsic molecular subtypes of BC are luminal A (ER^+^, PR^+^, and HER^−^, the most common), luminal B (ER^+^, PR^+^, and HER^+^), HER2-enriched (ER^−^, PR^−^, and HER^+^), and triple-negative (ER^−^, PR^−^, and HER^−^) (48). Triple-negative BC (TNBC) is the most difficult subtype to treat and the most lethal subtype amongst all BC subtypes (49–51).

**Figure 1:**
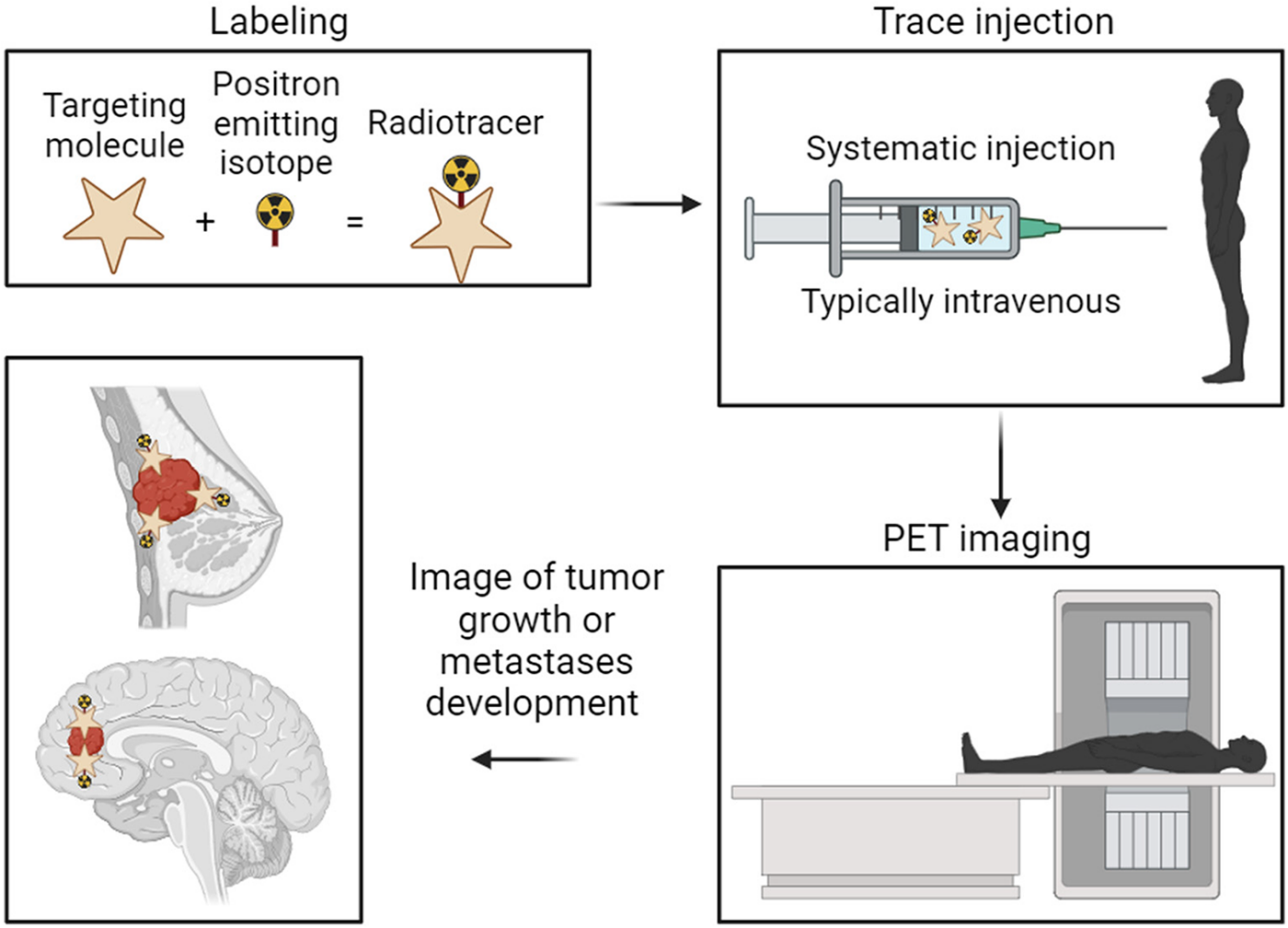
Representation of the basic principles and procedures in positron emission tomography (PET) imaging for cancer diagnosis. The targeting molecule is radiolabeled with the positron-emitting isotope. The now radiolabeled targeting complex is then introduced to the patient, typically through injection directly into the bloodstream. The isotope emits positrons within the patients’ system, allowing the PET camera to track the high-density locations of those positron emissions. Finally, an overall picture is generated showing the highest concentration locations of the radiolabeled tumor-targeting complex, thus showing the location of the tumor.

Radionuclides employed in BC therapy approaches can be categorized into diagnostic radionuclides and therapeutic radionuclides. Radionuclide-based imaging has been more frequently employed in diagnosing BC in recent years and is particularly important in ascertaining the extent of metastatic disease (52, 53). It offers indisputable advantages to the functional detection of BC through radionuclide-labeled small metabolic compounds for non-invasively illustrating the biological process of BC and radionuclide-labeled ligands/antibodies for specific ligand/receptor interaction-mediated targeting radionuclide molecular imaging of BC (54–56). One well-known clinical technique showing the advantage of radionuclides in cancer therapy is positron emission tomography (PET). As both a research and medical technique, PET is a functional imaging tool employing radiolabeled substances, such as glucose, whose function is to monitor the metabolic processes to detect tumors and search for metastases. The most popular clinical radionuclide used for detecting primary tumors and metastases is ^18^F-fluoro-deoxy-glucose (FDG) (Figure 1).

**Figure 2:**
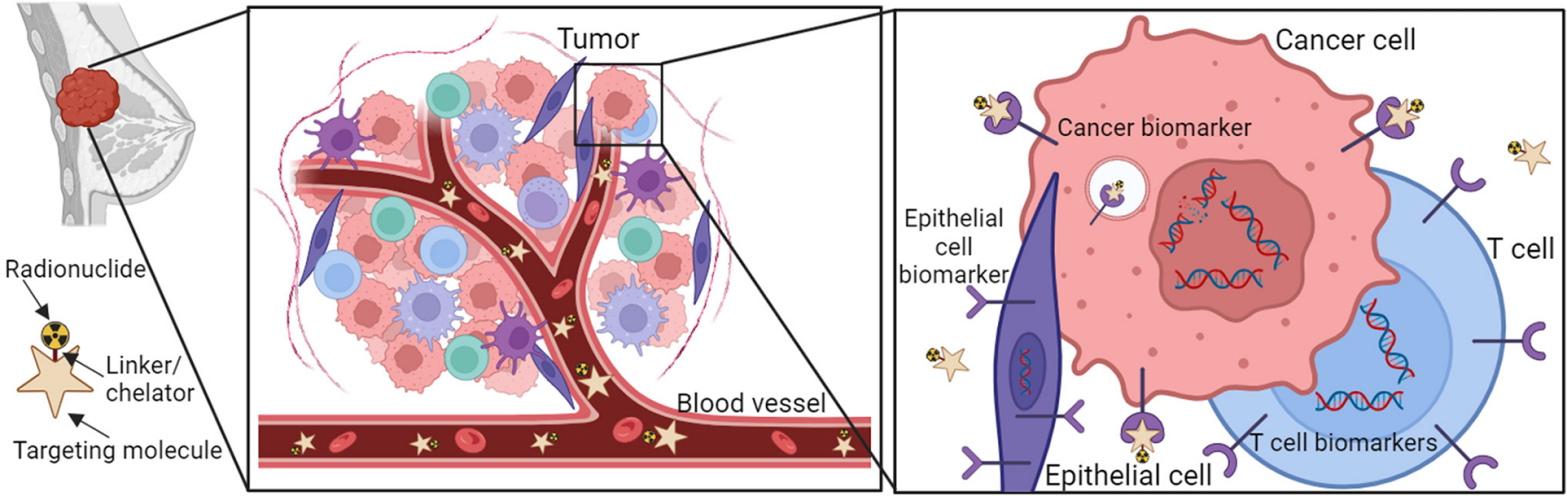
Schematic overview of receptor-targeting molecular imaging for BC. The molecular radionuclide structure consists of a ligand, linker, chelator, and radionuclide. Ligands that bind to the overexpressed receptors on BC cells can be coupled to chelator often through a linker. Chelators enable the labeling of ligands with radionuclides.

The principle of radionuclide-facilitated diagnosis of BC is illustrated in Figure 2. A common radionuclide cancer therapy is composed of three interconnected parts: A cancer cell-surface specific targeting molecule, a synthetic binding molecule that can specifically bind to the targeting molecule (called a linker), and a radionuclide-labeled chelator that is linked to the binding molecule (Figure 2) (57–59). All these three parts plus the specific surface marker on the cancer cells ensure the radionuclides target BC with high specificity and a high affinity, excluding potential off-target effects.

We have listed the commonly used radionuclides and their application in BC in Table 1. Amongst them, ^99m^Tc is the most popular and ideal imaging radionuclide because of its high target/non-target ratio and affordability (71–74).

**Table 1:** A list of radionuclides employed in the BC.

Radionuclides	Mode of decay	Physical half-life	E_max_ (MeV)	Application in breast cancers	Refs
^131^I	β	8.02 days	0.6	SPECT/PET imaging and targeted treatment	(60)
^177^Lu	β	6.73 days	0.5	Biodistribution and small-animal SPECT/CT imaging	(61)
^111^In	β^+^	67.2 h	0.245	SPECT/PET imaging	(62)
^18^F	β^+^	1.83 h	1.656	PET imaging	(63, 64)
^13^N	β^+^	9.97 h	1.30	PET imaging simplified for clinical applications	(65)
^15^O	γ/β^+^	122.266 s	1.73	PET imaging	(66)
^68^Ga ^99m^Tc	β^+^ γ	68 min 6.04 h	1.9 0.14	Imaging, including targeted, pre-targeted, and non-targeted imaging. SPECT	(14, 67–70)

## 3 Radionuclide treatment in breast cancer

**Figure 3:**
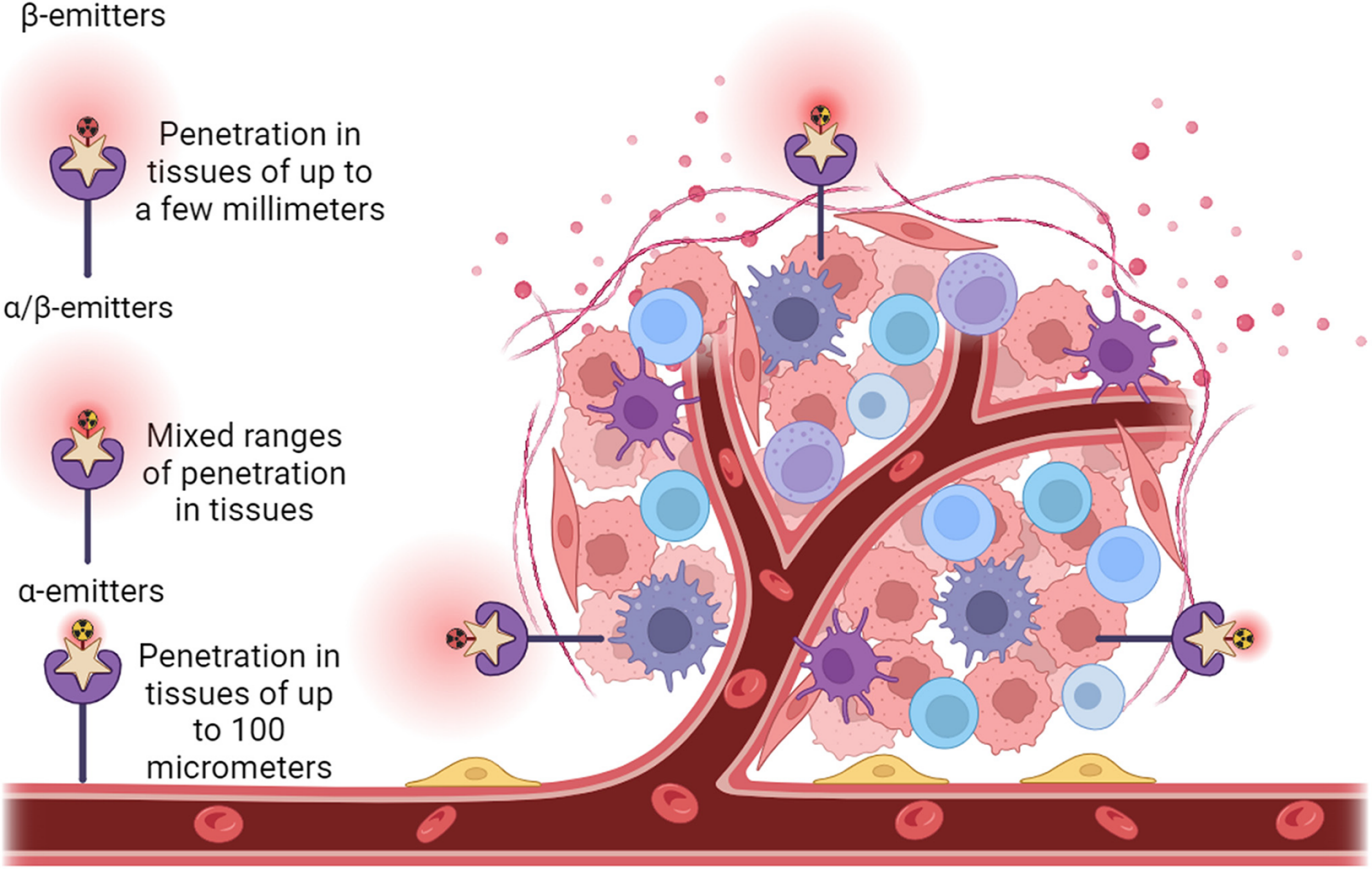
Administration of radionuclide carriers in the tumor tissue and their further accumulation via active targeting approach of a HER2^+^ tumor. Representative view of a tumor growing along blood vessels that is HER2^+^ (purple star receptors). Antibody structures (beige stars) that are radionuclide (radioactive yellow or red symbols attached to antibodies) carriers are introduced. The antibody carriers direct the radionuclides straight to HER2^+^ tissues such as the tumor, reducing ionizing damage to non-cancerous tissues. On the left of the tumor is a solely β particle emitter (red radioactive symbol), centered above the tumor is both and α and β particle emitter (red and yellow radioactive symbol), and to the right of the tumor is a solely α particle emitter (yellow radioactive symbol). Range and depth of tissue penetration of the respective radionuclides is shown as red clouds centered around the radionuclides.

As to the clinical radionuclide treatment, an optimal radionuclide therapy may provide therapeutic options that were not previously available for BC patients (Table 2). For effective treatment of BC, it is important to maximize tumor cell DNA damage and cytotoxicity while minimizing effects on nearby healthy tissue. The properties of radionuclides that support this therapeutic effect include a short half-life (85), linear energy transfer (86), toxicity (87–89), the range in tissue (2, 90, 91), *in vivo* stability (92, 93), tissue preference (85), the accumulation in tumors (90), the DNA damage scale (70, 94, 95), and the post-treatment clearance (96, 97). The double-stranded DNA damage caused by the selected particles emitted by a given radionuclide will determine the live or death of the targeted BC cells (69, 94, 98). Theoretically, double-stranded DNA breakage may only require the energy from a single alpha particle or multiple beta particles (99). Although the cytotoxicity of beta particles to cancer cells is much lower than that of alpha particles, alpha particles will generate much less toxicity than beta particles to the surrounding tissues of the tumor because of their short range (less than 100 micrometers), rendering alpha particles a focus for future use in clinical application (100). However, due to the increased penetration of beta particles, beta-emitting radionuclides are currently more frequently used for cancer treatment as they cause more widespread damage that can be somewhat constrained by penetration depth. For example, in 2009, the BC therapeutic agent trastuzumab (Herceptin, a humanized anti-HER-2/neu monoclonal antibody) was cross-linked with succinimidyl 3,6-diaza-5-oxo-3-[2-((triphenylmethyl)thio) ethyl]-8-[(triphenylmethyl)thio] octanoate (SOCTA) followed by labeling with the radionuclide ^188^Re; using a preclinical orthotopic mouse model, this ^188^Re-SOCTA-trastuzumab was administered intravenously to mice carrying tumors developed from BT-474 BC cells (HER2^+^). The results demonstrated that ^188^Re-SOCTA-trastuzumab accumulated much more in tumors than in normal tissues, suggesting that ^188^Re-SOCTA-trastuzumab can be a potential agent for targeted therapy (78). A combination treatment was employed using ^131^I radioactive iodine-conjugated antibodies to target the HER2 antigen to cause cancer cell death. The outcomes from the clinical trials demonstrated the safety and efficacy of this combined therapy for HER2^+^ BC (101). A recently developed targeted radionuclide theragnostic agent, ^131^I-GMIB-Anti-Her2-VHH1, has been tested for safety, biodistribution, radiation dosimetry, and tumor-imaging potential in the diagnosis and treatment of HER2^+^ BC (102). The results indicated that this agent could be a promising drug to image and treat HER2^+^ BC with much fewer side effects (Figure 3). Recently, Trastuzumab (Herceptin), a monoclonal antibody targeting HER2 receptors, has been covalently bound with 3-phosphonopropionic acid (CEPA) NP and labeled with ^225^Ac. ^225^Ac@Fe_3_O_4_-CEPA-trastuzumab has shown a high receptor affinity in a preclinical *in vivo* study in which the significant inhibiting potential of ^225^Ac@Fe_3_O_4_-CEPA-trastuzumab for BC was validated (103, 104).

**Table 2:** List of radionuclides employed in the treatment of BC.

Radionuclide	Mode of decay	Physical half-life	Formulation	E_max_ (MeV)	Application	Refs
^177^Lu	β	6.73 days	^177^Lu-bombesin-paclitaxel	0.5	For targeting EGFRs as a novel neoadjuvant brachytherapy for the treatment of locally advanced BC.	(75)
^153^Sm	β	46.50 h	^153^Sm EDTMP	0.8	A treatment for bone metastases.	(76)
^186^Re	β/auger	3.72 days	^186^Re-HEDP	1.1	A treatment for bone metastasis.	(77)
^188^Re	β/γ	17.00 h	^188^Re-SOCTA-trastuzumab	2.1	Treatment of inflammatory disease and cancer.	(78)
^213^Bi	β	45.61 min	Multiple chelators reviewed by Ahenkorah et al.	5.9	As targeted alpha-particle therapy.	(79)
^212^Bi	α/β	60.55 min	^212^Bi-MAA	6.1	For the development of cancer therapeutic agents for treating various neoplastic diseases.	(80)
^211^At	α	7.21 h	Multiple ligands reviewed by Guerard et al.	5.9	Treatment for cancer and first clinical trials.	(81)
^212^Pb	β	10.64 h	^212^Pb-CSPG4	0.6	For targeted pre-clinical and clinical use for the management and treatment of cancer.	(82, 83)
^224^Ra	α	3.63 days	^223^Ra-EDTMP	5.7	For the treatment of breast cancer bone metastases.	(84)
^131^I	β	8.02 days	^131^I-GMIB-HER2-VHH1	0.6	For targeted treatment of HER2^+^ breast cancers.	(60)

Radionuclide-labeled antibodies have been tested to improve the therapeutic efficacy and specificity (105–107). The programmed cell death ligand 1 (PD-L1), also named B7-H1 or CD274, is a key element of an immune checkpoint system and is essential for avoiding autoimmunity (108). PD-L1 is expressed on most cancer cells, tumor-associated macrophages (TAM), dendritic cells, activated T cells, as well as cancer-associated fibroblasts within the tumor microenvironment (109–111). PD-L1 can inhibit CD8^+^ T-cell effector function by interacting with programmed cell death 1 (PD-1) on the surface of T cells (112, 113). Antibodies against PD-L1 have been generated and evaluated in multiple clinical trials against BC, generating exciting outcomes for BC patients (114); perhaps more excitingly, combined anti–PD-L1 therapy with targeted radiotherapy has been shown to yield a higher therapeutic efficacy when compared to the antibody treatment alone (115). Notably, ^111^In [In]-BnDTPA-trastuzumab-NLS is another radiopharmaceutical agent with theranostic applications for imaging and auger electron radioimmunotherapy of HER2-positive BC (116).

The theranostics of lymph node metastasis is a major barrier to the successful treatment of BC and a key decision-maker in BC patients' prognosis. Notably, a recently designed nano nuclear drug (^68^Ga-NP-mAb or ^177^Lu-NP-mAb) displayed exceptional stability, considerable accumulation, and sustained retention in the lymph node metastases post-intratumoral injection. This agent not only significantly reduced the incidence of lymph node metastasis but also shrank the volumes of lymph node metastases as well without apparent toxicity in a mouse model (117). This is an example of how radionuclide-mediated therapy will open new avenues for the diagnosis and treatment of BC metastases. For TNBC, which has no ideal cell surface biomarkers available for targeted therapy, auger emitters have demonstrated an excellent therapeutic effect as long as they can be delivered directly into the nucleus proximal to DNA. The nuclear protein poly (ADP-ribose)-polymerase 1 has been reported as a possible target, but ideal inhibitors (PARPi) are not clinically available for current therapy of BC carrying the BC gene germline mutation (BRCA^mut^). A recent study employed a theranostic approach in a xenografted mouse TNBC model by radiolabeling a close derivative of the PARPi Olaparib (e.g., PARPi-01) with the auger emitters ^125^I, or [^125^I] PARPi-01. The results support the potential role of [^125^I] PARPi-01 in improving the use of radiation and radionclides to treat TNBC (118, 119).

Radionuclides such as radium-223 (Xofigo) (77, 119–121), strontium-89 (122–124), and samarium-153 EDTMP (125, 126) have been employed to treat BC bone metastases since their radioactive particles (α or β) are most likely to be absorbed in the setting of intensive bone turnover. To improve the efficacy of radionuclide therapy, radionuclides have been frequently combined with nanoparticles (NPs, 1–100 nm in diameter) in BC therapeutics due to their specific advantages for drug delivery. These include biocompatibility, low toxicity, high stability, excellent penetration ability, and tissue retaining efficiency (127–129). NP can be generated from any solid or liquid material, such as dielectrics, semiconductors, inorganic molecules, and organic molecules. To treat epidermal growth factor receptor (EGFR)-positive TNBC, gold NP (AuNP) was modified with polyethylene glycol (PEG) chains derivatized with 1,4,7,10-tetraazacyclododecane-1,4,7,10-tetraacetic acid (DOTA) chelators for conjugating with emitter ^177^Lu and with PEG chains linked to panitumumab (selectively binds to EGFR) for targeting TNBC cells with surface EGFR expression. Non-targeted ^177^Lu-NT-AuNP and EGFR-targeted AuNP (^177^Lu-T-AuNP) were subcutaneously administered into xenograft mouse models bearing EGFR^+^ MDA-MB-468 human BC tumors. The results demonstrated that ^177^Lu-T-AuNP is a potent radionuclide therapeutic agent for EGFR-positive TNBC therapy (130–132).

For early-stage cancer, antibodies targeting BC cell surface antigens such as CEA, MUC1, and L6 had been employed (68). Technitium-labeled anti-CEA Fab′ fragments showed 94% more sensitivity in breast tumors in a small clinical trial (133). ^90^Y-labeled humanized BrE3 antibody against MUC1 displayed a promising anti-tumor effect in a clinical trial (134, 135). The chimeric L6 antibody labeled with ^131^I also showed a considerable anti-tumor effect in clinical trials (136–138).

Chondroitin sulfate proteoglycan 4 (CSPG4), a highly glycosylated transmembrane protein, has recently been identified as a target for TNBC treatment due to its high expression on the surface of TNBC cells and its limited expression in normal tissues (more than six times lower than tumors) (139, 140). Therefore, we can use monoclonal antibodies recognizing CSPG4 conjugated to a radioactive isotope for radioimmunotherapy, which we achieve by targeting radiation directly and more specifically tumor cells, reducing non-specific exposure of normal cells to the radioactive isotope. Monoclonal antibody 225.28 specifically against CSPG4 was radiolabeled with ^212^Pb, allowing ^212^Pb-mAb 225.28 to specifically recognize TNBC cells and cause cell death *in vitro* and thus tumor reduction in a xenograft-bearing mouse model. These promising outcomes support ^212^Pb-mAb 225.28 as a potential therapeutic agent against TNBC (82).

Prostate-specific membrane antigen (PSMA) has been recently shown to be highly expressed on the cell surface of TNBC cells and adjacent endothelial cells, suggesting that PSMA can be a promising target for TNBC treatment. [^177^Lu] Lu-PSMA induced frequent apoptotic events in BT-20 and MDA-MB-231 tumor-associated endothelial cells, significantly limiting the proliferation of TNBC cells in the *in vitro* co-culture cellular models tested (141). Significant uptake of radiolabeled ligand [^68^Ga]Ga-PSMA was detected in BC stem cells expressing a high level of PSMA proteins on their cell surface (67). Furthermore, the hypoxic environment significantly promoted the uptake of radiolabeled ligand [^177^Lu] Lu-PSMA in MDA-MB-231 and MCF-7 cells (142). ^177^Lu has also been used to label tumor-targeting alkyl phosphocholine (NM600) for TNBC radionuclide therapy. ^177^Lu-NM600 has been shown to considerably extend the survival rate in syngeneic murine models bearing tumors developed from either 4T07 or 4T1 TNBC cells (143).

Mesothelin is a glycosylphosphatidylinositol-anchored cell-surface glycoprotein that is highly expressed in BC cells with a severely limited expression in normal tissues (144–146). A mesothelin-targeted thorium-227 conjugate, BAY 2287411 was tested for binding activity, radio stability, biodistribution, mode-of-action, and antitumor potency using an *in vitro* cellular model, an *in vivo* orthotopic model, and a patient-derived xenograft model. This demonstrated that BAY 2287411 treatment induces double-strand DNA breaks, apoptosis, and oxidative stress. It significantly decreases cell viability and shows a high antitumor potency. Biodistribution studies also suggested a specific uptake and retention of BAY 2287411 in tumors and not in normal tissues (147).

Fulvestrant (an endocrine therapy drug for BC) was labeled with radionuclide ^131^I to generate ^131^I-fulvestrant followed by an evaluation of its effect on BC cell viability and attenuation of the development of human BC and its toxicity to major organs in xenograft nude mouse models. ^131^I-fulvestrant is remarkably stable and shows a strong binding affinity to estrogen receptor-positive (ER^+^) MCF-7 cells (148). In addition, ^131^I-fulvestrant exhibited significant cytotoxicity in MCF-7 and MDA-MB-231 cells (ER^−^) and exerted a more pronounced suppressive effect on tumors derived from MCF-7 cells than from MDA-MB-231 cells. After ^131^I-fulvestrant was injected into nude mice intravenously, the distribution of radioactivity was tracked to ER expressing locations, and the majority of ^131^I-fulvestrant was confined to the tumors. ^131^I-fulvestrant could attenuate the proliferation of MCF-7 BC cells *in vitro* and inhibited the growth of tumors derived from implanted MCF-7 cells in nude mice whereas the toxicity of ^131^I-fulvestrant to the major organs of mice was mild and controllable. This renders ^131^I-fulvestrant a promising drug for BC treatment that combines the advantages of both radiotherapy and endocrine therapy (148).

In a newly-developed target alpha therapy, an anti-androgen receptor (AR)-targeted radiotherapy platform (Hu11B6) labeled with the alpha-particle emitting Ac-225 (^225^Ac-hu11B6) has been evaluated in murine xenograft AR-positive BC models. The results show a successful site-specific delivery of therapeutic Ac-225 to tumor tissues and effective, long-term, local tumor control (149). ^177^Lu labeled bombesin-poly (D, L-lactide-co-glycolide) acid (paclitaxel) NP display specific cellular uptake and high treatment efficacy in both *in vitro* and *in vivo* BC mouse models since bombesin can specifically recognize the gastrin-releasing peptide receptor, that is overexpressed on more than 75% of BC (75). While ^223^Ra has been evaluated to treat bone metastases in BC patients, in a mouse model, the administration of ^224^Ra significantly reduced the bone metastatic incidence from tumors developed from implanted MDA-MB-231(SA)-GFP human BC cells (84).

## 4 The challenges of radionuclide therapy for breast cancer

Growing evidence has suggested that radionuclide therapy holds great potential to robustly improve the treatment efficacy of BC, especially for imaging and therapy. However, one upfront challenge to be solved is the off-target effects and toxicity from radionuclides emitting beta particles, such as ^90^Sr, ^14^C, and ^210^Pb (100, 150). Although radionuclides emitting alpha radiation have a high linear energy transfer and can treat cancer rapidly with high efficiency, their half-life is generally much shorter. There may be a need to combine alpha and beta radiation emitters in a proper ratio to achieve the best therapeutic outcomes. We may also need to find a way to calculate the ratio of the radiation uptake between tumor tissues and surrounding normal healthy tissues. The implementation of radionuclide therapy also has to deal with the deep social fear of radioactivity (151).

Over the last three decades, drug delivery agents and methods have been intensively investigated to discover efficient and specific drug delivery protocols with limited or no off-target effects to improve the diagnostic and therapeutic outcomes of BC; these include peptides, small nanobody molecules, monoclonal antibodies, fragments of monoclonal antibodies, exosomes, and NP (152–156). However, amongst the aforementioned methods, one challenge for radiolabeled antibodies is the intra- and inter-tumor heterogeneity of their uptake by cancer cells, and imaging techniques with high resolution are required to show this heterogeneity. Certain situations may require two types of radionuclides to achieve the best imaging and therapeutic results, one radionuclide for imaging and another one for therapy (157). The antibodies' size limits their capability to penetrate tumors, directing the radiation emitted from radionuclides away from their targeted sites. As such, nanobodies have recently emerged as promising alternatives to robustly increase the capability of tumor penetration (158, 159). Although the resistance mechanisms to alpha particles are not significant, cancer cells may develop mutations to limit the specific delivery of radionuclides to cancer cells, such as the decreased expression of cancer cell surface antigens that are selected for targeting. Therefore, identifying more antigen and epitope candidates on BC cell surfaces for radionuclide-specific targeting will become increasingly indispensable to the elimination of tumors that have already acquired resistance to previous radionuclide therapy.

Thus far, there is not a medical constituency for radionuclide diagnosis and therapy, implying a need for a new specialty to provide the multidisciplinary training (in general oncology, radiation oncology, and nuclear medicine) necessary for safe, efficient, and effective administration of radionuclides to BC patients. Having expertise in both imaging and radionuclide dosimetry becomes extremely important for a medical physicist because the delivery of radionuclides and the distribution of radiation are critical for the successful treatment of BC. In addition, dosimetry calculations for the medical radioisotopes and daughters are still challenging given the many factors that need to be considered (160–164). It is necessary to accurately determine radiochemical purity and dosage as well as to account for both the parent radionuclide and associated decay daughters, as their relocation from the tumor site potentially places healthy tissues at risk. Promisingly, there is a novel method to minimize the release of the radioisotope daughters by encapsulating the radionuclides in exosomes (155) or by conjugating with nanobodies as we have currently designed in our laboratories. These exosomes carry a specific targeting peptide or nanobodies that can recognize the specific cell surface marker on BC cells allowing us to evaluate their anti-oncologic capability in pre-clinical animal models. There are still many additional tests that need to be done before being applied clinically, but novel treatment approaches are clearly needed for this challenging disease.

## 5 Perspective

Despite several radiopharmaceuticals being used for therapeutic targeting that have shown clinical value in many types of cancer and have been or will soon be approved and authorized for clinical use around in the world, radiopharmaceuticals do possess many side effects as well. Therefore, more research is required to establish the efficacy of therapeutic strategies; use in combination with other treatment modalities may result in better efficacy and reduced side effects. Hence incorporation of correlative biomarker studies is imperative to draw meaningful conclusions for individualizing critical therapeutic decisions that can be effectively generalized and implemented beyond the setting of this clinical trial. Additionally, testing the immune priming potential of radiation in combination with chemotherapy and/or immune checkpoint inhibitors will also provide a novel opportunity to induce immune modulation in BCs, which are largely considered to be poorly immunogenic.
